# Medical News and Miscellany

**Published:** 1899-06

**Authors:** 


### Medical News and Miscellany.

**The Brazos Valley Medical Society,** at its recent meeting in Marlin, Texas (May 9-11), elected the following officers for the ensuing year: President, Dr. I. P. Sessions, Rockdale, First Vice-President, Dr. W. P. Gilstrap, Wheelock, Texas; Second Vice-President, Dr. W. T. McKnight, Marlin, Texas; Treasurer, Dr. J. W. Hudson, Milano, Texas; Secretary, Dr. W. B. Briggs, Easterly, Texas (re-elected). The next meeting of the society will be held at Hearne, Texas, November 14, 1899. We present herewith several of the papers which were read at the Marlin meeting.

---

**Dr. A. N. Perkins,** long time Quarantine officer at Sabine Pass, has removed to Beaumont, Texas, and his son, Dr. W. P. Perkins, long resident at Bastrop, La., has joined him there.

---

**Death of Dr. D. Harris.**—Dr. Douglas Harris, of Whitney, Texas, one of the oldest and most beloved physicians in Texas, died at his home in Whitney, May 15, 1899. Dr. Harris was most exemplary in his life, and will be greatly missed. He was an old and zealous Mason, and Whitney Lodge testified their respect to his memory by most complimentary resolutions. Dr. Harris had been a subscriber to the JOURNAL from its first issue, fourteen years.

---

**A Case of Yellow Fever in New Orleans.**—One swallow does not make a summer; no more does one case of yellow fever make an epidemic; but the moral (and demoralizing) effect of the appearance of this one case in New Orleans is the same as if it were an epidemic. It has been thought sufficient to cause the Texas authorities to whack on the brakes and stop all trade and travel between that city and the great State of Texas, and as it is only the first of June, there is hardly a hope of a let up till frost, five or six months hence. Meantime, the people will have plenty of leisure. Mississippi has not quarantined; neither has Alabama nor Florida, but have sent inspectors to New Orleans. It would seem that where the first case of yellow fever is promptly recognized, as in this instance, it would be possible to destroy the focus of infection and thus prevent a spread; and we feel assured that prompt measures were taken to that end. Dr. Blunt's action in quarantining the City of New Orleans because of this one case may be the safest course, but is it wise? Is it justifiable? He could have done no more were the city ablaze with infection. The memorable epidemic of 1878 resulted from infection from Cuba brought in by the Emily B. Souder in May, but there had been a number of cases before the disease was recognized.

---

**Dr. R. H. Knolle** has removed from Cedar to Schulenburg, Tex. Dr. F. C. Ford from Nacogdoches to Houston.

---

**The** *St. Louis Medical Review* has been purchased by Dr. H. W. Loeb, who will edit and publish it in future.

---

**For Sale or Trade.**—A small property and a good practice in a rich country near the coast, central part; will sell on easy terms or trade for a place in West Texas or New Mexico. Address S. E. E., care Texas Medical Journal, Austin, Texas.

---

**Send Us Some Copies** of our January number. We will credit each sender with one month on his subscription for each copy sent. The demand for this edition was so great that we let go our file numbers rather than disappoint any one. The attraction was "Sanitary Needs of Texas."

---

**Dr. D. B. Anderson** has removed from Sherman to Brownwood and entered into co-partnership with Dr. W. B. Anderson of that city.

---

**The Alma, Alma, Mich.**—Dr. E. S. Pettyjohn, Vice-President of the Alma Sanitarium Company, has leased the Sanitarium for a number of years, and having introduced a number of improvements, will maintain the high character for excellence that institution has always borne. Texas patronage is soliciced. Write to Dr. Pettyjohn for details of the management, terms, etc.

---

**A "Thing of Beauty,"** and therefore, "a joy forever," is the I. & G. N. R. R. *Illustrator and General Narrator*. The first issue of this beautiful magazine—March, 1899—has reached us. The picture on the front cover is, in itself, a work of art, and is worth preserving. It is a beautiful colored lithograph in many colors. The magazine is copiously and well illustrated with scenes along the lines of that great through system, the I. & G.

N. R. R., which, connecting with other lines at Longview, traverses the entire State of Texas from Northeast to Southwest, and, connecting at Laredo with the Mexican National, constitutes a great through line to the City of Mexico. The work is intended to exploit the wonderful resources of Texas, which, in many parts of the State, are as yet scarcely touched, and, incidentally to advertise the great through line and especially its "high-flyer;" "through from City of Mexico to St. Louis" in lightning time. Write to D. J. Price, G. P. A., Palestine, Texas, for a sample copy and fuller information, mentioning the "Red Back."

---

**Dr. W. T. Davidson,** Assistant Surgeon Third Texas U. S. Volunteers, Fort Clark, Texas, has been ordered to report to General Lee at Havana for duty.

---

**Changes of Address:** Dr Wm. Osborne, from Rockdale to Bluffton; Dr. Julian McLemore, from Timpson to Crandall; Dr. M. L. Martin, from Galveston to Denton; Dr. R. L. Denman, from Durst to Angelina.

---

**The books** reviewed in this issue—all new and just out—are for sale at prices stated, less 20 per cent.; cash to accompany order. As there is but one of each order quick or you will get left.

---

**Sajou's Annual of Medical Science** for 1892-3-4-5-6, in neat cloth covers, and clean we offer at a big discount. There are five volumes in each set, and the price is $3 per volume. We will sell the sets at $5 each, or $1 per volume; those of 1892 at 75c per volume, cash with order.

---

**This number** completes vol. 14 of the Red Back.

---

**Here We Are:**—Yellow fever again. "Yellow fever is prevailing at Vera Cruz, Mexico, and is of a very malignant type. From May 1 to May 20 there were 148 cases, of which 67 proved fatal. New cases now develop at the rate of eight or ten per day with an average mortality of fifty per cent."—Telegram.

---

**College Consolidation.**—The Missouri Medical College and the St. Louis Medical College (Medical Department Washington University)havecon solidated, with Dr. H. H. Mudd, of the latter, as professor of surgery and clinical surgery, and dean of the faculty.

---

**Dr. G. C. Savage** has been elected Secretary of the Vanderbilt University. The Medical Department of the Vanderbilt is a great favorite with the Texas boys, and shows up a handsome list of Texas matriculants and graduates every year. Announcement of next session will appear, as usual, in our July number.

---

**Smallpox—Vaccinate.**—Chiles' Drug Store, Austin, Tex., has a supply of fresh glycerated vaccine virus on hand, and keeps it in an up-to-date "cold storage" compartment. Physicians in the interior will find it convenient to order it from Austin instead of from St. Louis or elsewhere.

